# Irrigation-coupled bipolar cautery unit: A practical, economical, and simple version

**DOI:** 10.4103/0970-0358.44932

**Published:** 2008

**Authors:** Shekhar Sharma, Altaf Gauhar Haji, D. K. Vijaykumar, A. K. Shaji

**Affiliations:** Department of Surgical Oncology, Amrita Institute of Medical Sciences & Research Center, Ernakulam (682026), Kerala, India

**Keywords:** Bipolar cautery, cost-effective irrigation-coupled, intravenous tubing, practical version, saline-linked electrocautery

## Abstract

Hemostasis is a fundamental principle of surgery for which electrocoagulation is universally used. Bipolar electrocautery has an advantage over monopolar electrocautery in terms of the precision of the extent of tissue coagulation and the lateral extent of thermal tissue injury. However, secondary to the thermal changes induced in the tissue due to electric current passage, there is charring of tissue, which adheres to the cautery tip. This, not only decreases its effectiveness, but also, by getting avulsed while removing the cautery tip from the surgical field, causes rebleeding and more trauma to the tissue. Irrigation of the surgical field during application of cautery reduces the charring effect, thereby improving the efficiency and efficacy. Irrigation-coupled electrocautery devices are available but are costly to acquire and maintain. We describe a simple and reliable version of an irrigation-coupled cautery device, which is of immense functional utility in our experience. It decreases the amount of charring of the tissue and its adherence to the bipolar forceps tips, thereby decreasing the frustrating loss of effectiveness and also increases the life of the bipolar forceps as cleaning needs to be less frequent. By virtue of its simplicity and cost-effectiveness, it can be used in almost all hospitals and situations.

## BACKGROUND

Hemostasis is a fundamental principle of surgery and electrocoagulation is the preferred method to achieve it in modern surgical era. Thermal changes induced due to cautery result in charring of tissue that then adheres to the cautery tip. This reduces the effectiveness of the cautery tip and due to avulsion, may cause more trauma and rebleeding in the tissues. This can be prevented by irrigation of surgical field, thereby improving both efficiency and efficacy of the applied electrocautery.[1–5] Historically, hot actual cautery (literal meaning of word ‘cautery’ burning / charring by application of something hot) or boiling oil were used to achieve hemostasis by forming a large tissue coagulum, which usually prevented bleeding until the entire dead mass sloughed away.[6]

The earliest such example can be found in ancient Egyptian writing, which describes the use of cautery in which the tip of a probe was heated and then applied to the tissue to produce coagulation, necrosis, or desiccation.[7]

Today, a more refined method to control bleeding is the application of regulated electric current (electrocautery). Electrocautery has now been in use for close to a century since its initial description by Doyen in his publication in 1906, followed by the development of the first practical electrosurgical generator in 1920 by Bovie.[7]

The use of electrosurgical energy has been the “gold standard” for hemostasis for quite some time now. It has diverse capabilities such as fulguration, precise vaporization, and coagulation of large vessels. Technological advances in performance and safety have positioned this device as a useful tool in a surgeon's armamentarium to the extent that use of electrocautery for hemostasis is now almost universal.

Bipolar electrocautery has now become an indispensable instrument for surgeons, not only because it provides precision control on the extent of coagulation and lateral thermal injury to adjacent tissue, but also because the path of flow of current from one tip of forceps to the other effectively eliminates the risks inherent with monopolar cautery.[8]

Consequent to this universal availability and use of bipolar electrocautery, surgeons are aware of the nuances of bipolar cautery forceps. It is frustrating to find the effectiveness of the bipolar cautery being lost so very often to the charring of tissues and its adherence to the forceps tip.

Saline-linked cautery units do away to a considerable extent with this nuance,[2] but are prohibitively costly to acquire and maintain. Among the not so well-equipped surgical theatres, considerable effort is directed at irrigating the field with syringes to decrease charring and / or very frequent cleaning of the forceps tip by an assistant to remove the charred tissue.

We describe a very economical and practical version of an irrigation-coupled cautery device, which is of immense functional utility in our experience.

## MATERIALS AND METHODS

The additional materials, other than a standard bipolar electrocautery forceps (Valleylab, US) needed for our version of a saline-coupled bipolar device, are a standard intravenous (IV) tubing set and 500 mL of 0.9% sodium chloride (saline) solution available in any hospital in abundance.

**Figure 1:**
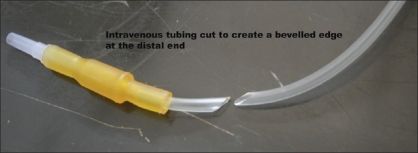
Intravenous tubing cut to leave a distal beveled edge

**Figure 2:**
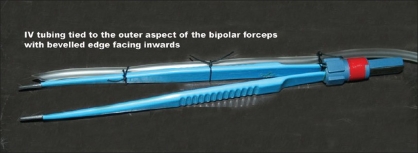
Intravenous tubing tied to the outer aspect of the bipolar forceps with beveled edge facing inwards

The pointed end of the IV tubing set is inserted into a 500 mL bag of 0.9% sodium chloride (saline) solution as usual. At the other end, the canula adaptor is cut off, leaving a beveled end of the plastic tube [Figure 1], which is tied close to the tip of the bipolar forceps tip using sterile surgical grade linen suture on the outer aspect of the forceps with the beveled end facing towards the forceps [Figure 2]. Bipolar forceps are now connected to a standard electrosurgical unit and the intravenous drip set is then primed with saline, the flow being adjusted as needed during surgery to obtain the appropriate tissue effect.

## RESULTS

We have been using this adaptation of a saline-linked bipolar device in our department for more than a year with gratifying results. Its advantages lie in the financial viability (additional cost to patient = Rs 57 only; US$ 1.42), ease of set up, and an absence of the need of an attentive assistant to either irrigate the surgical field or clean the bipolar forceps. During this year of use of this modification, in addition to more effective coagulation due to decreased charring, an added advantage is the precision of the extent of coagulation due to the thermal sink effect of the saline drop at the site of coagulation, leading to controlled heat generation and its even distribution.

Initial hiccoughs did appear, mainly in terms of adjusting the power settings for the cautery and the control of the flow at the cautery tip to achieve adequate coagulation without wasting much time. But once used to it, the setup, priming, and tuning were remarkably easy for all of us, requiring on an average about 15 minutes to assemble the modification prior to each use (10–25 minutes). A few important tricks we learned during our initial period of use of this device were: i) the beveled cut end of the drip set should be facing towards the forceps, ii) for best results, a drip rate of about 8–15 drops a minute is ideal and iii) most important for the modification to function as required, there should be a drop of saline bathing the tissue to be coagulated at the time of delivery of the current across the tips of the bipolar forceps.

## DISCUSSION

The coagulation process involves a sudden change in tissue characteristics that occurs within a narrow temperature range, closely following the pattern of the applied current [Table 1]. The high temperature associated with conventional cautery results in a charring of the tissue, smoking, and the development of a superficial blood coagulum plug that stops bleeding. However, this plug often cracks or is dislodged, resulting in intraoperative or postoperative rebleeding from the site. In bipolar cautery, tissues adhere only to the anode tip of the forceps due to the migration of negative polarized erythrocytes to the positive pole during coagulation.[5] Hence, coagulation by forceps will fail in time and the length of lost time will increase while the tips of the forceps will be damaged because of more frequent cleaning by the nurse or assistant.[2]

**Table 1:** Tissue changes at variable temperatures generated during electrocautery application[6]

*Temperature (°C)*						

	*33-44*	*44-50*	*50-80*	*80-100*	*100-200*	*>200*
Effects
Visible	None	None	Blanching	Shrinkage	Steam ‘popcorn’	Carbonization cratering
Delayed	Edema	Necrosis	Sloughing	Sloughing	Ulceration	Larger crater
Mechanism	Vasodiatation, Inflammation	Disruption of cell membrane	Collagen denaturation	Dessication	Vaporization	Combustion of tissue hydrocarbons

Irrigation with saline and in some papers isotonic mannitol (although we have not used it) during the operation results in a considerable reduction of charring and adherence of coagulated tissues and blood clots to the forceps tips in the coagulation of both arteries and veins.[3] These solutions have been used in injectors for irrigation or in cups for wetting the tips of forceps, both of which imply that the attention of assistants would be diverted from more important issues.

On theoretical grounds, the power needed to coagulate a surface point depends upon the impedance of the circuit (from the cautery tip to the ground plate in monopolar and from one cautery tip to the other in bipolar cautery). This impedance depends upon a number of factors, the main ones being the distance between the two electrodes and the conductance of the tissue forming the circuit. Thus, power needed while using bipolar cautery can be as low as 5% compared to that needed while using monopolar cautery,[6] This difference in power explains the apparent paradox as to why bipolar cautery works under water, whereas monopolar cautery causes the accumulated liquid to boil under the same conditions.

The earliest examples of automatically irrigated bipolar forceps are provided by King *et al.* in 1972 and Dujovny *et al.* in 1975.[2,5] Dujovny *et al.* described a model using bipolar forceps with an inbuilt suction channel and an irrigating pump with a self-designed mechanism to couple it to the cautery unit to provide irrigation to the forceps tip.[2]

This is similar to the commercially available irrigation-coupled cautery devices which work by coupling radiofrequency energy from a standard electrosurgical generator with saline irrigation to conduct thermal energy. Saline used as a conductive fluid at the tip of the device, cools the tissue surface and prevents the surface temperature from exceeding 100°C. This is in contrast to conventional electro surgery where temperatures reach in excess of 300°C.[4] Saline-linked cautery has reduced or minimal charring and hence, greater depths of tissue coagulation with controlled precision are possible in both monopolar and bipolar modes.[1,4,7,9]

Rampini and colleagues are to be credited with the first description of a low-cost, self-irrigating modification of irrigation-coupled cautery units in 1997.[1] In the article, authors have described a method to attach a tube to the outer aspect of bipolar forceps, which can then be used in a fashion similar to the modification suggested by us. Rampini *et al.* used sterlisable glue to attach a silastic tube, which was then attached to a luer lock to allow for the attachment of an irrigating unit to the outer aspect of bipolar forceps. There are two main concerns with this design: i) by the act of gluing, one pair of bipolar forceps is perforce dedicated to irrigation coupling, which may not be acceptable to all surgeons sharing the equipment, ii) inadvertent shearing force on the tubing risks the avulsion of the outer coating of bipolar forceps glued to it, which can render a costly pair of forceps useless. Both the disadvantages are avoided in the modification suggested by us. Additionally, the availability of fine silastic tubing as used by Rampini *et al.* (inner diameter 0.76 mm; outer diameter 1.65 mm), an attachable luer lock, and sterlisable glue is difficult and adds to the cost in our set-up.

Interestingly, thermoprotective effects of irrigation-coupled cautery devices on vital structures in the vicinity, although claimed by many and for long, have been studied only recently and that too in animal models.[10] In their elegantly designed and conducted study on rat sciatic nerves, Donzelli *et al.* were the first to show objectively that simultaneous irrigation of the cautery field resulted in decreased neuronal injury and faster recovery.

The authors were able to conclude on the basis of their observations, that the thermo protective effects of simultaneous irrigation was not due to a decreased temperature rise, but due to faster thermal recovery of tissues. The fact remains open for discussion as to whether this thermal recovery was due to simultaneous irrigation alone or additionally due to irrigation continuing after switching off the bipolar current under test conditions.

Commercially available devices use a special pair of bipolar forceps that have a channel built into it along with a cautery-coupled irrigation pump. This delivers a steady, regulated flow of irrigation to the cautery tip from within when it is activated (hence, the term “coupled”).

The main advantage of such “*off the shelf*” devices is the inbuilt nature of design, precluding the need for assembly prior to each use. Additionally, the inbuilt channel along with a cautery-coupled irrigation pump provides a steady, controlled irrigation to the forceps tip from within. Another advantage is that the irrigation is linked to the cautery activation, delivering fluid only when required.

This is in contrast to the modification suggested by us where an average of 15 minutes (10–25 minutes) is needed prior to each use to assemble the modification and to prime it. In our modification, fluid is delivered onto one tip, thereby necessitating a short learning curve to ensure a drop of fluid hanging before the initiation of cautery current, which in turn mandates that fluid flow be set at a predetermine pace throughout the surgery, causing wasting of fluid and a nuisance of a wet surgical field.

However, all technological advancements come at a price and hence, such irrigation-coupled bipolar devices are financially unaffordable except for a minority of resource-rich, affluent hospitals.

Although initially perceived by colleagues in our department to have decreased handling and to lack precision, all our surgical colleagues concluded otherwise after two or three session of use. As the tubing is tied on the external aspect, it does not, in any way, interfere with either the ease of handling or precision of cautery application. On the contrary, as mentioned above, a better precision was observed due to a thermal sink effect.

Our modification, though not a path-breaking description, is an affordable version that can be used in most of the hospitals, which already have a standard electrocoagulation unit.

## CONCLUSIONS

In our experience, this device is a reliable method of preventing charring at the tip of cautery forceps. Thus, it is a reliable, cost-effective and easy-to-implement modification. In our experience and opinion, it renders the bipolar cautery unit more user- and patient friendly and also prolongs the life of the bipolar forceps tip.
